# Experimental and Theoretical Investigations of Direct and Indirect Band Gaps of WSe_2_

**DOI:** 10.3390/mi15060761

**Published:** 2024-06-06

**Authors:** Yingtao Wang, Xian Zhang

**Affiliations:** Department of Mechanical Engineering, Stevens Institute of Technology, Hoboken, NJ 07030, USA

**Keywords:** WSe_2_, photoluminescence, band gap, optoelectronics

## Abstract

Low-dimension materials such as transition metal dichalcogenides (TMDCs) have received extensive research interest and investigation for electronic and optoelectronic applications. Due to their unique widely tunable band structures, they are good candidates for next-generation optoelectronic devices. Particularly, their photoluminescence properties, which are fundamental for optoelectronic applications, are highly sensitive to the nature of the band gap. Monolayer TMDCs in the room temperature range have presented a direct band gap behavior and bright photoluminescence. In this work, we investigate a popular TMDC material WSe_2_’s photoluminescence performance using a Raman spectroscopy laser with temperature dependence. With temperature variation, the lattice constant and the band gap change dramatically, and thus the photoluminescence spectra are changed. By checking the photoluminescence spectra at different temperatures, we are able to reveal the nature of direct-to-indirect band gap in monolayer WSe_2_. We also implemented density function theory (DFT) simulations to computationally investigate the band gap of WSe_2_ to provide comprehensive evidence and confirm the experimental results. Our study suggests that monolayer WSe_2_ is at the transition boundary between the indirect and direct band gap at room temperature. This result provides insights into temperature-dependent optical transition in monolayer WSe_2_ for quantum control, and is important for cultivating the potential of monolayer WSe_2_ in thermally tunable optoelectronic devices operating at room temperature.

## 1. Introduction

Since graphene was first exfoliated in 2004, two-dimensional (2D) materials [1,2,3,4,5,6,7,8], with their atomically thin crystalline structure, have received extensive research attention due to their unique atomically thin structure and novel physical properties. They have unique electronic [1,2,3,7,9], optical [4,7,10,11,12], mechanical [13], and energy harvesting [14,15,16] properties for enabling novel 2D devices that make them ideal materials and platforms for future information technology devices [14,17,18,19,20]. Among all 2D materials, transition metal dichalcogenides (TMDCs) materials, in the family of MX_2_ (M: Mo, W; X: S, Te, Se), have been discovered as a new class of semiconductors for atomically thin electronics and optoelectronics. They presented superior and intriguing properties [16,21,22,23,24,25] in optical, thermal, and electrical, such as prominent band structure [26], enhanced figure of merit [22], and semiconducting behavior [4], which distinguishes them from graphene. This makes TMDCs materials an ideal candidate for the next-generation optical, thermal, and electrical devices.

Specifically, there is a particular interest in monolayer (1L) MX_2_, which have been identified as direct band gap semiconductors and emerged as new optically active materials for developing novel 2D-material-based light emitters and absorbers. Experimental evidence of direct band gap in 1L MX_2_ was obtained from the observation of strongly enhanced photoluminescence (PL) caused by the indirect-to-direct band gap transition when MX_2_ were thinned to 1L, while its bulk form has an indirect band gap. The indirect-to-direct band 1L MX_2_ has been believed to be a common property. Although the common TMDCs materials—MoS_2_’s optical and semiconducting properties have been studied extensively [4,27,28,29]—the investigation of the emerging optoelectronic material WSe_2_ has received much less attention.

On the other hand, 2D WSe_2_ has emerged as a promising candidate for optoelectronic devices due to its high quasi-ballistic transport [30]. It has been shown that 1L WSe_2_ exhibits a direct band gap (K-K) with strong photoluminescence [31]. This has led to an increasing interest of not only discovering WSe_2_’s band gap from the fundamental aspect, but also its optoelectronic device applications such as light-emitting diodes, photodetectors, and lasers. An indirect band gap in WSe_2_ will limit their application in optoelectronic devices. If at another temperature WSe_2_ exhibits an indirect band gap behavior, it will be an important discovery for optoelectronic devices’ design. Thus, discovering the band gap properties of 2D WSe_2_ and the temperature-dependent trend is of fundamental importance and also lays immediate guides to the development of flexible thin film optoelectronic devices, thereby revolutionizing both fundamental optical and semiconducting properties in materials and application technologies in optoelectronic devices.

Temperature modulation of WSe_2_ will change its lattice constant and therefore change its band structure. This will further modulate the energies of the conduction band (CB) minima and valence band (VB) maxima for the material. If the energy difference of the direct and indirect band gaps is small, it will be possible to achieve a crossover from one to the other using temperature modulation.

Temperature and power-dependent PL tests of monolayer WSe_2_ have been conducted to investigate the variations in PL peaks, and the linear relationship between PL peak intensity and temperature or laser power has been observed and discussed, elucidating underlying principles such as competitions between localized and delocalized exciton emissions [32] or the opening of additional non-radiative relaxation channels [33]. In this study, we endeavor to examine the relationship between temperature and PL peak position and elucidate its underlying mechanisms through Density Functional Theory (DFT) calculations.

While the thermal, electrical, and optical properties of 2D WSe_2_ have been previously studied, to date, there is no experimental research reported on the temperature-dependent band gap behaviors in 2D WSe_2_. Therefore, it is needed to conduct a comprehensive experimental band gap investigation of WSe_2_ in the 2D atomic-layered form to discover the intrinsic band gap behavior with a temperature tuning.

In this article, we demonstrate a drastic change of WSe_2_’s band gap with a temperature modulation from 140 K to 600 K. Experimental, computational, and theoretical investigations have been conducted in WSe_2_’s band gap behaviors—a key parameter in the optoelectronic properties. We have found that band gap is tunable by temperature, and at the room temperature, 1L WSe_2_ is at the transition boundary between indirect and direct band gap. Experimental work demonstrates the phenomena, and computational work by DFT further confirms a temperature-dependent indirect-to-direct transition in 1L WSe_2_, with a theoretical analysis from these results. This work not only demonstrates a tuning mechanism for WSe_2_’s band gap, but also sheds light on engineering next-generation optoelectronics.

## 2. Materials and Methods

We prepared the monolayer WSe_2_ (1L WSe_2_) sample by the scotch tape mechanical exfoliation method from the WSe_2_ bulk crystal from 2D Semiconductors Inc., Phoenix, AZ, USA on 1 cm × 1 cm SiO_2_ (285 nm)/Si substrates. Silicon substrates with an oxide layer SiO_2_ of a thickness of 285 nm are being used because this thickness can provide the best optical contrast for us to distinguish between 1L, 2L, and few-layer WSe_2_ under the optical microscope. In total, 1L and few-layer WSe_2_ flakes were found using an optical microscope, and their thicknesses were further confirmed using Raman spectroscopy and an atomic force microscope [34].

Temperature-dependent photoluminescence (PL) experiments were performed on Raman spectroscopy equipment (RENISHAW inVia Raman Microscope system), with the 1L WSe_2_ on SiO_2_/Si sample standing on a temperature controllable platform (Linkam Stage THMS 600). The temperature-controllable platform allows for both a heating up and a cooling down of the sample in order to explore the properties in a broad temperature range, from 140 K to 600 K, with a step of 20 K. The 1L WSe_2_ sample was protected in a pure N_2_ environment to prevent sample oxidation and degradation during the heating and cooling process. A 514 nm green laser was used for Raman testing, and a power of 200 μW was employed to conduct PL and Raman tests.

In order to fully understand the evolution of electronic states, we have conducted a density functional theory (DFT) calculation for a 1L WSe_2_’s band gap based on temperature. We used ABINIT, which is an open-source program for implementing density functional theory by solving the Kohn–Sham equations of electron potential. The unit cell is defined as hexagonal, with the angle between the lattice direction a and b is 60 degrees. The lattice parameters used in the calculation are a = b = 3.2–3.5 Angstrom and c = 129.63 Angstrom, with the two Se atoms defined at the (0, 0, 0) and (0, 0, 0.0258) locations of the unit cell, and the W atom defined at the (1/3, 1/3, 0.0129) location of the unit cell. The values of a and b are varied in order to simulate the temperature-dependent lattice expansion, with a = b = 3.283 Angstrom at 273 K. The value of c is chosen to be ten times larger than the bulk WSe_2_ in order to simulate single layer electronic band property. For the calculation, we used local density approximation (LDA). The convergent tolerance of the potential is set to be 1 × 10^−15^ and the energy cutoff is set to be 20 eV. We calculated the first 20 electronic bands and presented them in Section 3. We select the k-vector trajectory of Γ → M → K → Γ, with Γ = (0, 0, 0), M = (0.5, 0.5, 0), and K = (1/3, 2/3, 0) in the first Brillouin zone.

## 3. Results

**Figure 1:**
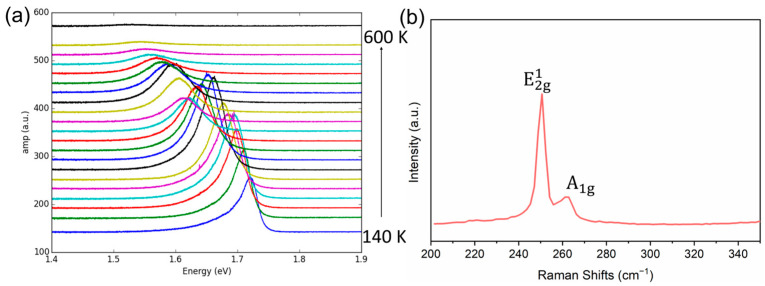
(**a**) Renormalized PL spectra of 1L WSe_2_ from 140 K to 600 K, at a photon energy range from 1.4 eV to 1.9 eV. (**b**) The Raman spectrum of monolayer WSe_2_.

Figure 1a shows the optical spectra of the PL signal’s intensity amplitude *I* as a function of the photon energy *E* at a broad temperature range from 140 K to 600 K.

The temperature-dependent PL curves present that at the lowest temperature of T = 140 K, the PL peak possesses the highest photon energy of 1.715 eV, along with a sharp PL peak (small linewidth). As the temperature increases, the PL peak shifts towards a lower energy range, with the PL peak intensity amplitude maximized at around 0 degree Celsius (T = 273 K). Above T = 273 K, as the temperature increases, the PL peak intensity amplitude starts to decrease and eventually disappears at around T = 600 K.

Figure 1b shows the Raman spectrum of monolayer WSe_2_. The monolayer’s characteristics can be confirmed by comparing the intensities of the $E_{2g}^{1}$ and A_1g_ peaks, as well as the absence of the $B_{2g}^{1}$ peak around 308 cm^−1^ [35,36].

The PL signal of peak curves can be fitted to a symmetric Lorentzian line shape function, as in the following:

(1) $$I=\frac{I_{0}∆E^{2}}{{\left(E-E_{0}\right)}^{2}+∆E^{2}}$$

where *I*_0_ is the PL peak intensity amplitude (a.u.), *E*_0_ is the PL peak position (eV), and Δ*E* is the PL peak linewidth (eV). This Lorentzian fitting function helps to extract the values of PL peak intensity amplitude, peak position, and peak linewidth for each PL peak.

**Figure 2:**
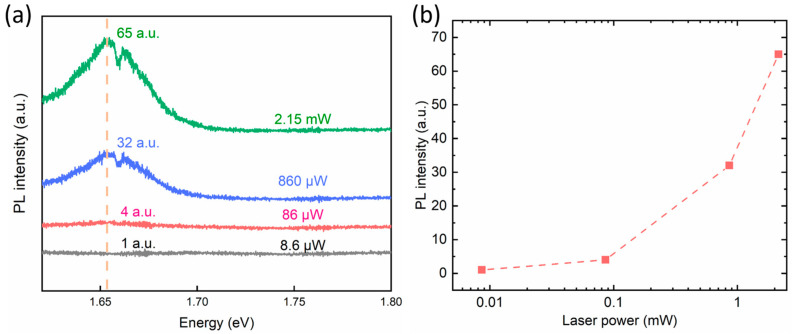
(**a**) The power-dependent PL spectra at 8.6 μW, 86 μW, 860 μW, and 2.15 mW, respectively. (**b**) The power-dependent PL intensity at about 1.65 eV.

Despite the precise temperature control provided by the instrumentation, capable of mitigating temperature deviations induced by laser heating, and our deliberate efforts to maintain consistent testing powers during experimentation, we conducted power-dependent PL tests to elucidate the influence of varying laser powers on PL intensity. As depicted in Figure 2b, at the scale of 0.1 mW, the impact on intensity remains confined within a narrow range, indicating the effectiveness and accuracy of our temperature testing procedures.

**Figure 3:**
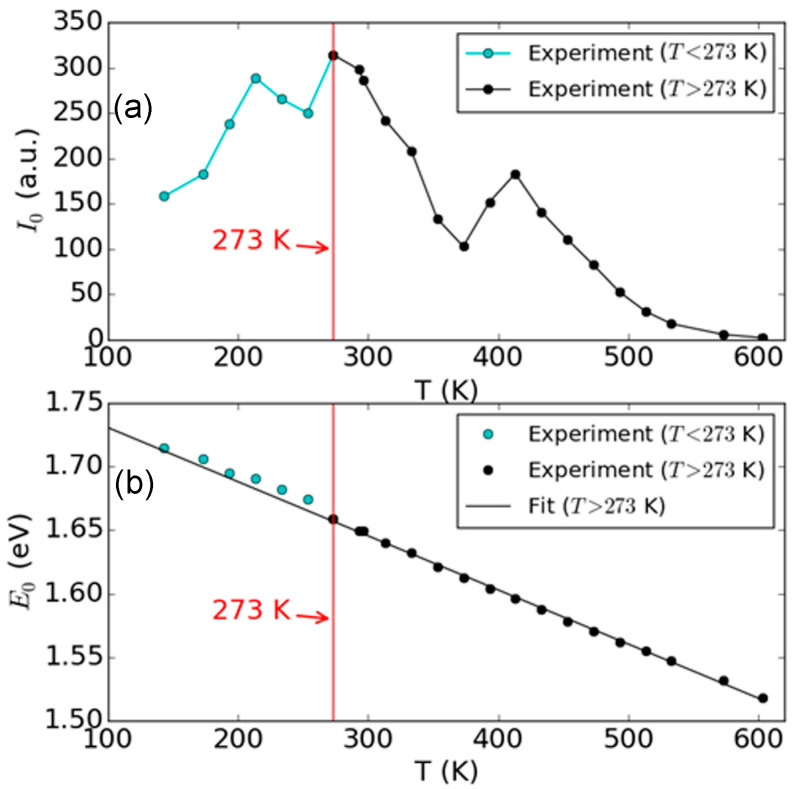
Extracted PL parameters from Lorentzian fitting of 1L WSe_2_ as a function of temperature. (**a**) Peak amplitude *I*_0_ and (**b**) peak position *E*_0_. The vertical red lines denote the transition of PL evolution at T = 273 K, corresponding to (**a**) the maximum of *I*_0_, and (**b**) the location where the *E*_0_*–T* slope has changed. The red lines indicate the transition from the indirect band gap (Γ K to K for T < 273 K) to the direct band gap (K to K for T > 273 K) PL.

Figure 3 shows the extracted PL peak intensity amplitude and the PL peak position as a function of temperature, from the Lorentzian function fitting. From the fitting results of PL peak intensity amplitude *I*_0_, we can observe two different regions, which are separated by T = 273 K, where a maximum of *I*_0_ is located at. In Figure 3, we marked this separation by a vertical red line at T = 273 K. This observation indicates that there is a change of the PL mechanism at 273 K, and this transition is from an indirect band gap PL (T < 273 K) to a direct band gap PL (T > 273 K). For PL peak position *E*_0_, it is clearly observed that there is a monotonous decrease with temperature (Figure 3b). And in addition to its monotonous decrease with temperature, we also observed a slight deviation from the linear extrapolation at a low temperature range (T < 273 K), which is also marked by the separation line. To present this difference, all the experimental results below 273 K are marked by cyan circles, and the data above 273 K are marked by black circles. In Figure 3b, we also plot the linear fit line for the data where T > 273 K and extrapolate it between 100 K and 600 K. The extracted slope is $k=-4.26\times 10^{-4}$ eV/K, which is primarily due to the thermal expansion of the 1L WSe_2_ crystal lattice and the resulting change of band gap.

**Figure 4:**
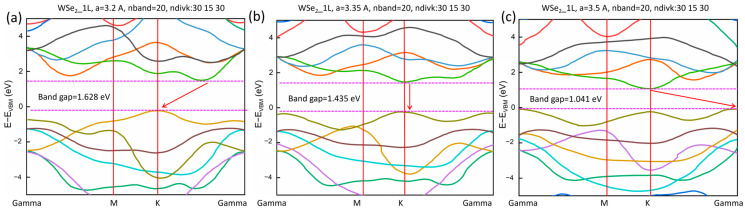
DFT-calculated band structure for 1L WSe_2_ with different lattice parameters. (**a**) a = 3.2 A, corresponding to indirect band gap with K Γ to K relaxation, (**b**) a = 3.35 A, corresponding to the direct band gap with K to K relaxation, and (**c**) a = 3.5 A, corresponding to indirect band gap with K to Γ relaxation. The band gaps are (**a**) 1.628 eV, (**b**) 1.435 eV, and (**c**) 1.041 eV.

In order to understand the impact of temperature’s effect and thermal expansion on 1L WSe_2_ and explain the temperature-dependent behavior of its PL and band gap, we have conducted density functional theory (DFT) calculations of the electronic band structures of 1L WSe_2_ at various lattice constants to simulate temperature-dependent lattice expansion and temperature-dependent band gap. Figure 4 shows the calculated first 20 electronic bands for 1L WSe_2_ along the Γ → M → K → Γ trajectory for three conditions: (a) a = 3.2 Angstrom, (b) a = 3.35 Angstrom, and (c) a = 3.5 Angstrom. The details of DFT parameter definitions and simulation steps are included in the Section 2. For the smallest lattice parameter, a = 3.2 Angstrom in Figure 4a, the maximum of the valence band (VBM) is at the K point, while the minimum of the conduction band (CBM) appears between the K and Γ point (K Γ). This will lead to indirect band gap PL process because the photon-excited electrons will primarily relax to the K Γ point in the conduction band, and the created holes will aggregate at the K point in the valence band, leading to the major electron–hole annihilation events from K Γ to K. When the lattice parameter is increased to a = 3.35 Angstrom, as shown in Figure 4b, the K point will become the CBM, which means the PL will be dominated by a direct band gap from K to K. And because this process satisfies both energy and momentum conservations, the probability of electron–hole annihilation is maximized, and the PL intensity will reach its peak. As the temperature increases, the lattice of 1L WSe_2_ will further expand. As shown in Figure 4c, at a = 3.5 Angstrom, the VBM will still happen at the K point, but the CBM will be changed from the K point to the Γ point, again leading to an indirect band gap PL. The occurrence of the three different regimes at their corresponding temperatures will be discussed below.

**Figure 5:**
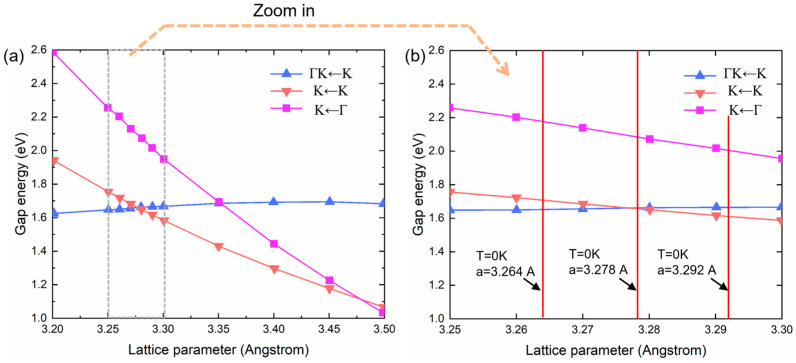
DFT calculated band gaps of 1L WSe_2_ between different conduction band maximum (CBM) and valence band minimum (VBM). (**a**) Complete relationship between gap energy and lattice parameter, showing energy crossing between direct band gap relaxation (K to K, pink) and two indirect band gap relaxation (Γ K to K, blue, and Γ to K, purple). (**b**) Zoomed band gap to lattice parameter dependence, with marked temperature extracted from the PL experiment.

Figure 5 summarizes the band gap energy for the three different scenarios from Figure 4 as a function of the lattice parameter a from 3.2 to 3.5 Angstrom. The main feature happens between a = 3.25 Anstrom and a = 3.3 Angstrom, where the evolution of the direct band gap PL from K to K point intersects with the indirect band gap PL from Γ K to K point, with the intersection taking place at a = 3.277 Angstrom. In order to identify their corresponding temperatures, we take the room temperature lattice parameter of WSe_2_ as a_0_ = 3.278 A [37]. For the thermal expansion of WSe_2_, we take the X-ray diffraction data from Ref. [38] as $a=3.264+4.717\cdot 10^{-5}\times T$, with a in Angstrom and T in Kelvin. In Figure 5b, we mark the three characteristic lattice parameters at different temperatures: a = 3.264 Angstrom corresponding to T = 0 K, 3.278 Angstrom corresponding to T = 300 K, and 3.292 Angstrom corresponding to T = 600 K. The lattice parameter at 300 K agrees well with the intersection of the Γ K to K gap and the K to K gap, meaning that the direct-to-indirect gap transition will happen at around the room temperature. This prediction agrees well with the experimental observations in Figure 3, and will serve as a quantitative support of this main conclusion in the work.

## 4. Discussion

From the experimental data and the DFT calculation result, we are able to calculate the thermal expansion coefficient of 1L WSe_2_. We first take the *E*_0_–T slope in the direct band gap regime (T > 273 K) in Figure 3b, ($k=-4.26\times 10^{-4}$ eV/K), which should be an accurate reflection of band gap change because there is only one PL path available (K→K). We then take the DFT calculated K→K band gap evolution from Figure 5b, as $l_{K\to K}=-3.42$ eV/Angstrom. The thermal coefficient α is calculated as $\alpha =\frac{k}{a_{0}l_{K\to K}}=3.8\times 10^{-5}$, or $1.25\times 10^{-4}$ Angstrom/K. This is more than twice the reported value for bulk WSe_2_, which is $1.45\times 10^{-5}$, or $4.72\times 10^{-5}$ Angstrom/K for the a-axis, in the reference Murray et al. [38]. A similar increase in α has also been reported from bulk graphite to 1L graphene [39], and from bulk to 1L transition metal dichalcogenides such as MoS_2_ [40,41] and MoSe_2_ [42,43]. It is worth noting that the PL that has been used in this work provides a simple and direct approach to studying the thermal properties of layered materials via linking to the electronic band structure. Micro Raman spectroscopy is another approach that is able to sensitively measure the temperature dependence of phonon modes in order to extract the thermal expansion coefficient of 2D materials. However, the data obtained from Raman spectroscopy are much more difficult to interpret, since the frequencies of the specific phonon modes are more complicated to calculate as compared to electronic band structures. In addition, sometimes there could also be a large difference in Raman-determined thermal expansion coefficient from different modes. For example, Kumar et al. [42] have reported a nearly ten-fold difference in α between the out-of-plane $A_{1g}$ mode and the in-plane $E_{2g}^{1}$ shear mode in 1L and 2L MoSe_2_, where Zhang et al. [41] have reported a consistent α for the $A_{1g}$ and $E_{2g}^{1}$ modes in 1L MoS_2_. For PL, the emitted photon energy is closely dependent on the minimal band gap of the material, which can be accurately calculated for a given lattice constant.

We now discuss the temperature evolution of *I*_0_ and *E*_0_ in Figure 3. In Figure 3a, *I*_0_ increases with temperature for T < 273 K, which is attributed to the change from an indirect band gap to a direct band gap. For T > 273 K, *I*_0_ decreases with temperature. This is mainly due to the increased relaxation rate of electrons during their scattering with lattice, which will determine the population of electrons at the CBM and the holes at the VBM during the excitation relaxation process. The excited electron hole pairs can annihilate by scattering with the lattice, and the energy will be relaxed to the lattice without emitting a photon. In Figure 3b, the deviation of *E*_0_ in the low temperature regime (cyan circles) from the linear extrapolation is likely due to the flattened band structure when the K→K direct gap and the ΓK→K indirect band gap energies degenerate and the resulting change in density of states. This may lead to more electronic states slightly higher than the CBM and create photon emissions with a larger energy. This observation is different from prior PL reports of 1L-WSe_2_, where a monotonic increase in PL intensity was measured as the temperature was decreased [32,33]. We attribute the difference to a better sample quality in our case, which quantitatively matches with the DFT calculation for the temperature boundary of direct-to-indirect bandgap transition.

For the PL spectra below 273 K, the line shapes are asymmetric, with a larger lobe for energy below the peak compared with the energy above the peak. This likely suggests the appearance of an additional PL peak at a lower energy from the main peak. It is worth noting that at a low temperature (<0 K) the 1L WSe_2_ becomes an indirect band gap material. However, because the K→K direct gap is very close to the ΓK→K indirect band gap, both cases may contribute to the PL spectra. As a continuation of the direct band gap light emission for T > 273 K, we attribute the main peak of the PL spectra for T < 273 K to the K→K direct gap, and the additional low-energy PL peak to the ΓK→K indirect band gap. The result is in contrast to a few other previous reports where lower-energy peaks are observed with a difference in photon energy from the main PL peaks [32,33]. In their reports, the lower-energy peaks are attributed to disorder-induced local excitations. In our results, the absence of lower-energy peaks suggests a defect-free sample condition for our PL measurements. We also point out that a finite exciton binding energy should be considered as a consequence of Coulomb interaction, and this energy can be different between direct and indirect bandgap transitions because of the different spatial confinement with different wavenumbers [44,45]. However, in 1L-WSe_2_, this binding energy difference has been reported to be 0.03 eV (theo) and 0.08 eV (exp) [45], both much smaller than the energy difference of the lower-energy peaks in the prior reports [32]. The binding energy difference is too difficult to resolve in our results because of the relatively large PL linewidths.

## 5. Conclusions

In conclusion, we have systematically investigated the direct and indirect band gaps in 1L WSe_2_ at a broad temperature range, experimentally and theoretically. Furthermore, 1L WSe_2_ is shown to exhibit drastic increase in PL intensity from 140 K to 600 K. Specifically, we have revealed the nature of direct-to-indirect band gap transition in 1L WSe_2_ in the room temperature range. At room temperature, 1L WSe_2_ is at the transition boundary between indirect and direct band gaps. This result is also verified using DFT at different temperatures and lattice constants. This phenomenon is mainly attributed to the increased relaxation rate of electrons during their scattering with lattice, which will determine the population of electrons at the CBM and the holes at the VBM during the excitation relaxation process. Then, the K→K direct gap is changed to the ΓK→K indirect band gap, and this leads to more electronic states that are higher than the CBM and creates photon emissions with a larger energy.

Moving forward, band structure and bandgap engineering with temperature can be used as a method to change the mobility of carriers in 1L WSe_2_. In addition, increased PL emission properties make temperature-heated/cooled or -strained/compressed 1L WSe_2_ a promising candidate for next-generation optoelectronic devices. In addition to emission properties, future absorption studies at various temperatures will provide promising insight into the potential use of 1L WSe_2_. The findings from this study provide key information for WSe_2_’s fundamental physical properties and its potential applications in thermally tunable optoelectronic devices at room temperature.

## Data Availability

Data are available upon request.
